# The Rare Association of Tetralogy of Fallot and ALCAPA

**DOI:** 10.21470/1678-9741-2019-0139

**Published:** 2019

**Authors:** Alexandre Noboru Murakami, Gabriela Guimarães Baston, Mariana Ribeiro Rodero Cardoso, Carlos Henrique de Marchi, Ulisses Alexandre Croti

**Affiliations:** 1 Serviço de Cirurgia Cardíaca do Norte do Paraná, Universidade Estadual de Londrina (UEL), Londrina, PR, Brazil.; 2 Pediatric Cardiovascular Surgery Service of São José do Rio Preto - Hospital da Criança e Maternidade de São José do Rio Preto (FUNFARME) - da Faculdade de Medicina de São José do Rio Preto (FAMERP), SP, Brazil.

**Table:** 

Abbreviations, acronyms & symbols	
ALCAPA	= Anomalous left coronary artery from the pulmonary artery
PCICU	= Pediatric cardiac intensive care unit
PDA	= Patent ductus arteriosus
RVOT	= Right ventricular outflow tract
TOF	= Tetralogy of Fallot

## Clinical Data

Infant, female, diagnosed at four months of age with congenital heart disease in the Northeast region of Brazil. At 1 year and 7 months old was referred to our service for further clinical investigation and possible surgical treatment.

On arrival, presented with history of cyanosis and dyspnea on activities of daily living and feeding, weighing 9,5 kg. During physical examination presented regular general condition, central and peripheral cyanosis and saturation around 70%. Presence of systolic murmur 2+/6+ predominantly at the upper left sternal border. Clear lung sounds.

## Electrocardiography

**Fig. 1:**
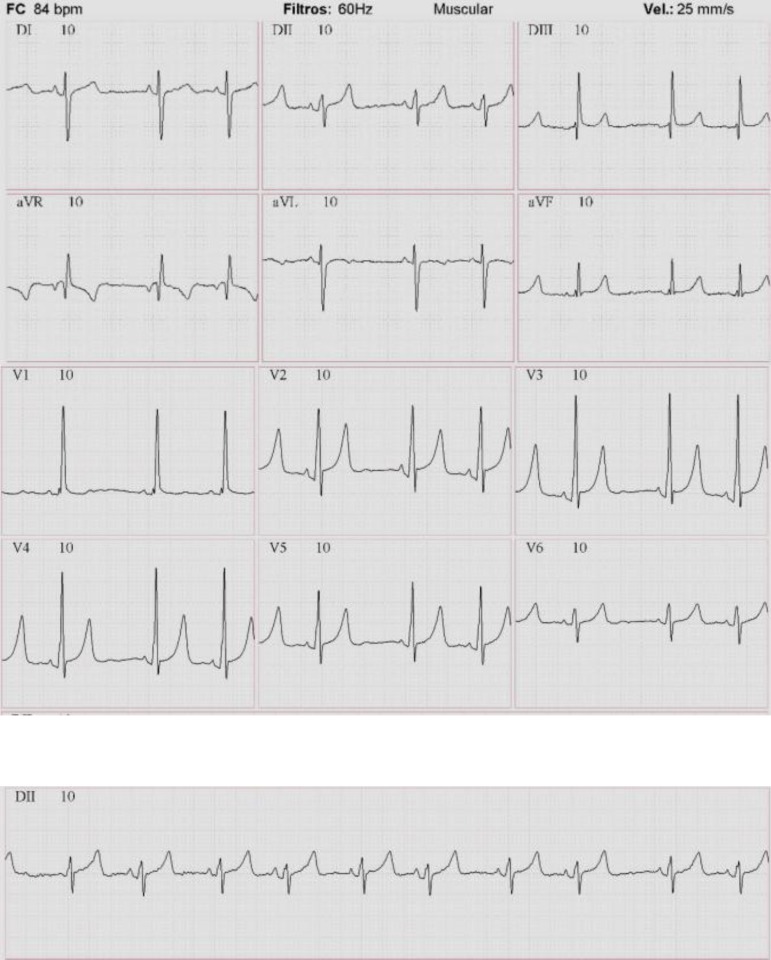
Electrocardiography showing sinus rhythm and right ventricular hypertrophy.

Sinus rhythm, heart rate: 84 beats/min, SAQRS +150º, and right ventricular hypertrophy (Figure 1).

## Chest Radiograph

**Fig. 2:**
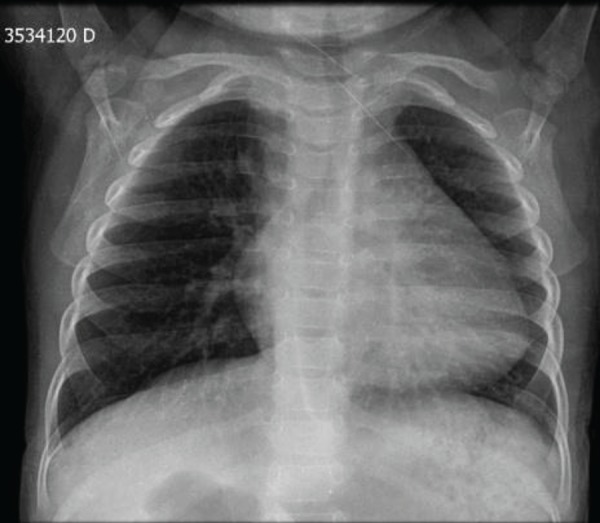
Chest radiograph showed increased cardiac area and normal pulmonary vascular tissue, not characteristic of TOF.

Cardiac enlargement with cardiothoracic ratio = 0.58. Suggestive of right aortic arch. Pleuropulmonary space unchanged (Figure 2).

## Echocardiography

Tetralogy of Fallot with subvalvar pulmonary stenosis with a peak gradient of 61 mmHg. *Ostium secundum* atrial septal defect (2 mm). Left coronary artery originating from the pulmonary artery. Infra-aortic innominate vein. Right aortic arch. Normal function of the right ventricle. Normal left ventricular contractile function from a global and segmental point of view.

## Computed Tomography Angiography

**Fig. 3:**
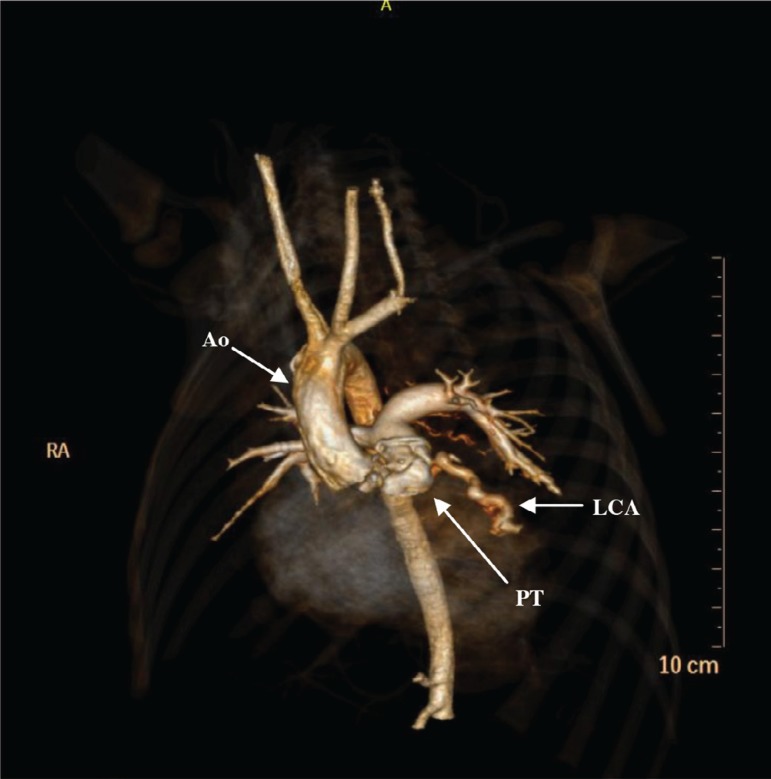
A 3D volume-rendered computed tomographic angiography showing anomalous origin of the left coronary artery from the pulmonary trunk and not from the aorta. The dilated left anterior descendant coronary artery (LAD) can also be seen. Ao=aorta; LC=left coronary artery; PT=pulmonary trunk

*Situs solitus* in levocardia, all connections were concordant. Tetralogy of Fallot (TOF). Anomalous left coronary artery from the lower margin of the pulmonary trunk, with dilation and tortuosity of the anterior descending coronary artery, as shown in Figure 3.

## Diagnosis

Approximately 5% of patients with TOF will have an anomalous coronary artery crossing the right ventricular outflow tract (RVOT)^[1]^, therefore, it is mandatory to evaluate the coronary artery during investigation.

As the echocardiogram showed left coronary artery originating from the pulmonary trunk, a computed tomography angiography was requested for coronary arteries evaluation (Figure 3).

Anomalous left coronary artery from the pulmonary artery (ALCAPA) is a rare congenital heart defect that occurs more often as an isolated defect. It is rarely associated with other cardiac defects such as ventricular septal defect, patent *ductus arteriosus* (PDA), TOF or coarctation of the aorta^[2,3]^.

Surgical correction is the gold standard therapy for ALCAPA and direct reimplantation of the anomalous artery into the aorta is the most frequent surgical technique^[4]^. In this case, surgery was indicated as soon as the diagnosis of ALCAPA with TOF was discovered.

## Operation

Transsternal median thoracotomy approach, heparinization with 4 mg/kg and careful cannulation of the aorta and vena cava were performed. Hypothermia at 20º Celsius, 212 minutes of cardiopulmonary bypass, including cross-clamping time of 147 minutes and circulatory arrest of 22 minutes.

Anterior and transverse pulmonary trunk opening, locating the left coronary ostium in the posterior wall of the pulmonary trunk and excising a button of the pulmonary artery wall around the coronary ostium.

An opening was made in the left posterolateral portion of the aorta and direct implantation of the left coronary button was performed into the aorta with polydioxanone 7-0 continuous sutures. The pulmonary trunk was reconstructed with a bovine pericardium patch.

Tetralogy of Fallot was corrected through disinsertion of the pulmonary valve, which was bicuspid, preserving the pulmonary annulus. After incision of the anterior wall of the right ventricular outflow tract, an infundibular resection was performed. Bovine pericardium patch was used to enlarge the right ventricular outflow tract.

The ventricular septal defect was closed with bovine pericardium patch using 6-0 polypropylene sutures through the right atrium and ventricle.

After surgery, the patient was stable and transferred to the pediatric cardiac intensive care unit (PCICU) for postoperative care.

The patient was treated for pneumonia, remaining 15 days in PCICU, 18 days in the pediatric ward (33 days of total hospitalization time).

**Fig. 4:**
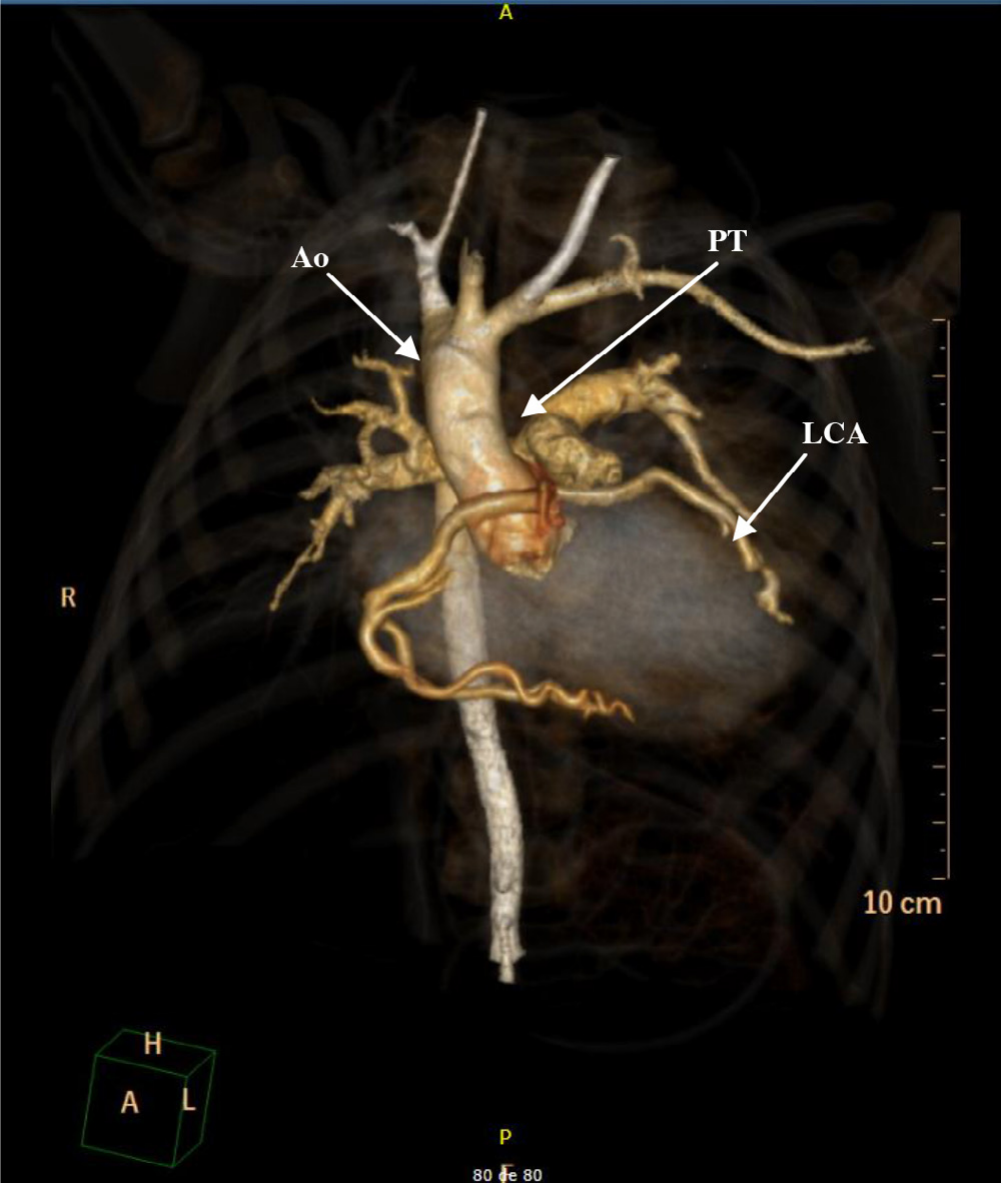
Postoperative 3D volume-rendered CT angiography showing reimplantation of the left coronary artery to the aortic root. Ao=aorta; LC=left coronary artery; PT=pulmonary trunk

Angiography prior to discharge showed excellent surgical outcome, as illustrated in Figure 4.

**Table:** 

Authors' roles & responsibilities	
ANM	Substantial contributions to the conception or design of the work; or the acquisition, analysis, or interpretation of data for the work; final approval of the version to be published
GGB	Substantial contributions to the conception or design of the work; or the acquisition, analysis, or interpretation of data for the work; final approval of the version to be published
MRRC	Drafting the work or revising it critically for important intellectual content; final approval of the version to be published
CHM	Drafting the work or revising it critically for important intellectual content; final approval of the version to be published
UAC	Drafting the work or revising it critically for important intellectual content; final approval of the version to be published
